# Not So Stable Angina: Single Coronary Artery Disease

**DOI:** 10.7759/cureus.21625

**Published:** 2022-01-26

**Authors:** Sittinun Thangjui, Harshith Thyagaturu, Ahmad Morshed, Hiroo Takayama

**Affiliations:** 1 Internal Medicine, Bassett Healthcare Network, Cooperstown, USA; 2 Cardiovascular Services, Bassett Healthcare Network, Cooperstown, USA; 3 Department of Cardiothoracic Surgery, Columbia University Irving Medical Center, New York, USA

**Keywords:** unroofing procedure, congenital anomalies of coronary arteries, coronary anomalies, anginal chest pain, stable angina, single coronary artery

## Abstract

A single coronary artery (SCA) is a rare congenital anomaly that can be incidentally found as a part of ischemic heart disease or angina workup. A modified Lipton classification is used to categorize the disease. The majority of diseases do not need surgical correction, with the exception of a few conditions. This report presented the case of a 49-year-old man who presented with stable angina with a single coronary artery arising from the right coronary sinus with an intraseptal course of the left main coronary artery. This is categorized as RII-S in the modified Lipton classification and is considered a high-risk anomaly. He underwent an unroofing procedure to decompress the left coronary artery with a resolution of symptoms.

## Introduction

Single coronary artery (SCA) was initially described by Lipton et al. in 1979 [1]. The combined incidence of SCA was approximately 3.3% of all coronary anomalies and 0.024-0.095% of the population [1,2]. A single coronary artery was classified as a potentially serious coronary anomaly along with an anomalous coronary artery from the opposite sinus (ACAOS) of Valsalva, coronary fistula, and coronary artery arising from the pulmonary artery [2]. Surgical intervention may be beneficial in selected patients. This case report describes a patient with stable angina with a rare diagnosis of a single coronary artery.

## Case presentation

**Figure 1:**
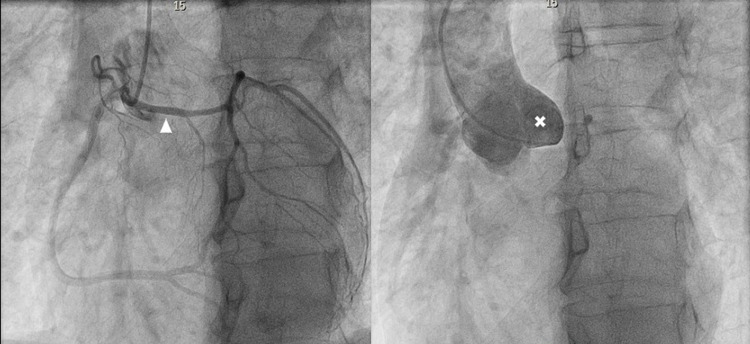
Coronary angiography Coronary angiogram showing single coronary artery arising from right coronary cusp with anomalous left main coronary artery (arrow head) originate from right side. Left coronary cusp contains no ostia and had no flow after injection of dye (x).

**Figure 2:**
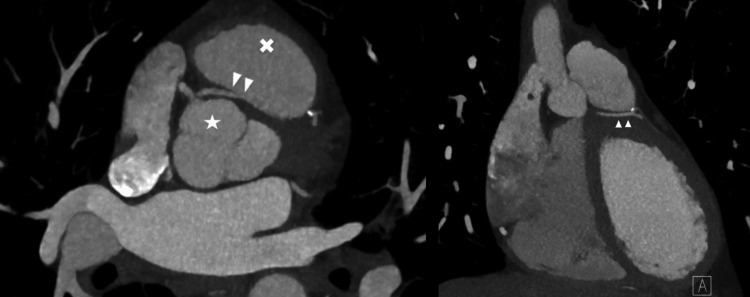
Coronary computed tomography angiogram findings of single coronary artery Coronary computed tomography angiogram showing single coronary artery arising from right coronary cusp (star). The anomalous left coronary artery (arrowhead) travels between the right ventricular outflow tract (x) and the aorta.

**Figure 3:**
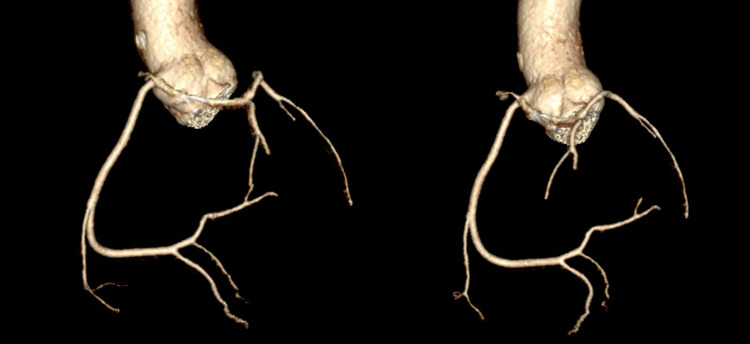
Coronary computed tomography angiogram with 3D reconstruction of the single coronary artery Coronary computed tomography angiogram with 3D reconstruction showing the single coronary artery arising from the right coronary cusp then separated into the right coronary artery and left coronary artery.

A 49-year-old white man presented with dyspnea and chest pressure on exertion. The chest pressure was consistent with typical angina, described as a centrally dull, aching, non-radiating pain with shortness of breath associated with exercise and resolved at rest. The patient had a past medical history of tobacco dependence and chronic obstructive pulmonary disease. On examination, his vital signs and cardiovascular and respiratory exams were benign. High sensitivity troponin I was negative on arrival and three hours apart. Electrocardiography showed sinus bradycardia with a right bundle branch block without new ST-T changes. Transthoracic echocardiography showed preserved left ventricular ejection fraction without regional wall motion abnormality. Stable ischemic heart disease was suspected. A nuclear stress test with regadenoson showed an abnormal study with borderline apical ischemia and a left ventricular ejection fraction of 49%. Coronary angiography and aortography showed that right coronary artery (RCA) and LM arise from a single ostium at the right coronary sinus (Figure 1). Coronary computed tomography angiography (CCTA) confirmed the anomalous origin of the left coronary artery (LCA) from the right coronary sinus with an acute angle at the origin with a transeptal course between the right ventricular outflow tract and the aorta before bifurcating into the left anterior descending coronary artery (LAD) and left circumflex coronary artery (LCX) (Figures 2, 3). There was a subtle calcification of the origin of the LAD, causing mild focal stenosis. This anomalous is categorized as RII-S by modified Lipton’s classification. Low-dose aspirin and atorvastatin were started to treat atherosclerosis.

The patient underwent unroofing procedure started by separating the main PA from the aorta. The space behind the main PA was identified and intraventricular septal muscle overlying the left main was divided until the LM merged to the epicardial arteries. The PA was then reanastomosed back. This procedure was intended to decompress the anomalous LM. The patient tolerated the procedure well and was discharged within 5 days. Followed up echocardiography showed normal ejection fraction and chest pressure on exertion symptoms resolved. 

## Discussion

A single coronary artery was categorized by the modified Lipton’s classification into three major groups [2]. Group I is a true SCA with a normal course of a single coronary artery (LCA or RCA) that arises from a normal coronary sinus followed by the other vessel originating distally from the normal artery. R and L were used to differentiate the origin of the right and left coronary sinus, respectively. Group II described large SCAs arising from either right or left coronary sinus, then separated into two major coronary arteries, LCA and RCA, and subclassified the group by the path of the abnormal artery related to the aorta and PA into A (anterior to PA), B (between the aorta and PA), P (posterior to the aorta), S (intramuscularly through the septum, intraseptal), and C (combined type). Group III described a patient with three arteries (RCA, LCA, and LAD) originating from a single coronary artery that arises from a single coronary sinus. Patients with SCA can be asymptomatic and commonly diagnosed as a part of an ischemic heart disease workup. The diagnosis is made by either coronary angiography, CCTA, or cardiac magnetic resonance (CMR) imaging [3,4]. Coronary angiography is considered the gold standard, but requires experience to identify the course of the anomalies. Cardiac magnetic resonance imaging and CCTA give more information in terms of identifying the surrounding structures related to coronary anomalies. Single coronary artery was less frequently associated with sudden cardiac death (SCD) compared to other types of coronary anomalies but the risk of SCD increases in right coronary sinus origin and interatrial course of LCA (between main pulmonary artery and aorta) or intraseptal course such as this patient [5]. The proposed mechanism of SCD explains that enlargement of PA and aortic root during exertion causes compression of the coronary artery course between the great arteries. However, this phenomenon does not always happen. Other high-risk factors are slit-like/fish-mouth-shaped orifices, acute angle takeoff, intramural course, and hypoplasia of the proximal coronary artery. European Society of Cardiology guidelines in 2020 recommended non-pharmacological functional imaging (i.e., nuclear studies, echocardiography, or CMR with physical stress) in patients with coronary anomalies to confirm/exclude myocardial ischemia. If positive for typical angina symptoms with imaging findings confirming the myocardial ischemia in a matching territory related to the anomalies, surgery is recommended [4]. The American Heart Association also recommended surgery in SCA with typical ischemic symptoms and positive stress imaging as a class I recommendation and a class IIa for those without symptoms [3]. However, the type of surgery recommended for the patient is still debatable because the benefit of surgery on long-term outcomes is still unclear [6]. The coronary artery bypass graft is not recommended in a patient without significant stenosis due to the risk of competitive flow from native vessels causing graft failure. Surgical correction is more commonly performed in SCA with interarterial and intraseptal courses. Reported surgical techniques for SCA with RII-S modified Lipton’s classification include pulmonary root mobilization and modified Lecompte maneuver; supraarterial myotomy by unroofing the artery into the right ventricular cavity; unroofing of the intraseptal LCA by circumferentially transecting and extending the right ventricular infundibulum using autologous pericardium [6-8]. In this case, simple unroofing of LM from the interventricular septum without pulmonary artery relocation was performed with a good outcome. This adds to the list of surgical techniques to correct this rare condition. As an increasing number of cardiac imaging is being performed, the incidence of coronary anomalies and SCA are expected to increase. Further studies are needed to determine the benefit of surgery correction compared to medical management.

## Conclusions

Single coronary artery disease is a rare disease with a lesser risk of sudden cardiac death compared to other types of coronary anomalies. This is commonly found in a patient as part of an ischemic heart disease workup and is unlikely to require further surgical intervention. However, surgical correction may be needed in high-risk group patients with interarterial and intraseptal courses of the anomalous left main artery.
